# Factors Associated with Medical and Health Talent Agglomeration at the City Level in China: Economic, Social, and Ecological Perspectives

**DOI:** 10.3390/healthcare14203417

**Published:** 2026-10-13

**Authors:** Lei Zhang, Lingmin Shao, Cheng Hu, Dayong Zhang

**Affiliations:** 1 School of Economics and Management, Zhejiang Sci-Tech University, Hangzhou 310018, China; 2025211104010@mails.zstu.edu.cn (L.S.); 2025221105004@mails.zstu.edu.cn (C.H.); 2 School of Medicine, Hangzhou City University, Hangzhou 310015, China

**Keywords:** medical and health talent, talent agglomeration, spatial autocorrelation, panel regression, multidimensional associated factors

## Abstract

**Highlights:**

**What are the main findings?**

- It extended the previously established multidimensional framework for analyzing the agglomeration of medical and health talent (including density, scale, intensity and equilibrium) to the longitudinal urban level.
- Urban hospital beds were identified as the most robust and consistently positive factors associated with medical and health talent agglomeration across different dimensions of talent agglomeration.
- Economic factors demonstrated nonlinear association and substantial heterogeneity across agglomeration dimensions and regions.

**What are the implications of the main findings?**

- Talent attraction policies should move beyond uniform approaches toward region specific strategies integrating economic, social, and ecological conditions.
- Strengthening medical resource capacity should be considered as a fundamental regional strategy for attracting and retaining medical and health talents.

**Abstract:**

**Background:** Medical and health talent (MHT) is a core strategic resource for safeguarding public health. Understanding the factors associated with MHT agglomeration is essential for improving healthcare workforce planning and optimizing the allocation of health resources. **Methods:** Using panel data from 285 prefecture-level cities in China spanning 2004–2019, this study extended the previously established multidimensional framework for analyzing MHT agglomeration encompassing density, scale, intensity, and equilibrium to a longitudinal urban setting. Spatial autocorrelation analysis and a two-way fixed effects panel model were used to examine economic, social, and ecological factors associated with MHT agglomeration. Robustness tests, endogeneity checks, and heterogeneity analysis were also conducted. **Results:** The spatial autocorrelation analysis reveals that the spatial distribution of MHT exhibits a generally random and dispersed pattern. Urban hospital beds per 1000 population maintained a significant positive association across all model specifications, representing the most robust factor associated with talent agglomeration. Sci-tech expenditure exhibited nonlinear association, while ecological factors displayed pronounced dimensional heterogeneity and regional differentiation. **Conclusions:** MHT agglomeration is associated with factors across multiple dimensions, with hospital bed capacity showing a particularly consistent association.

## 1. Introduction

The Healthy China strategy is a vital component of China’s current economic and social development, and medical and health talent (MHT) is central to its implementation. As global competition in scientific and technological innovation intensifies, MHT remains a core strategic resource for advancing medical technology, improving public health services, and safeguarding population health and well-being. The efficiency of its spatial allocation directly affects the overall effectiveness of the national healthcare system and coordinated regional development. Since China’s reform and opening-up, MHT has become highly concentrated in eastern coastal regions and major cities. Recent studies on the spatial distribution of primary care resources and high-quality medical services have widely documented this pattern [1,2,3]. Although this uneven distribution has improved innovation efficiency, it has also exacerbated interregional inequalities in medical and health resources, limiting equitable access to high-quality medical resources across regions.

Compared with traditional labor migration, the location decisions of highly skilled medical and health professionals are more complex. These professionals consider not only conventional economic incentives, such as remuneration and benefits, but also career development opportunities, family quality of life, children’s education, and the urban ecological environment. However, the convergence of local policies in the current competition for talent reflects an insufficient understanding of the factors associated with talent agglomeration. Many regions still rely excessively on economic instruments, such as one-off relocation subsidies and salary bonuses. They give less attention to building long-term attractiveness by improving social services and the ecological environment, which limits the precision and sustainability of talent policies. Therefore, identifying the key factors associated with MHT agglomeration and determining how their associations differ across agglomeration dimensions are important research priorities for optimizing the spatial allocation of talent and developing more targeted policies.

Although existing studies have examined economic, social, and ecological factors influencing talent mobility, several issues remain insufficiently explored at the city level. First, prefecture-level cities are important local labor markets and administrative units for allocating healthcare resources, providing public services, and implementing talent policies in China. Analyses conducted only at the national or provincial level may therefore obscure substantial differences among cities within the same province [4]. Second, although previous studies have examined the distribution of physicians and healthcare resources across Chinese cities [5,6], many relied on a single indicator or a limited set of conventional indicators, such as physicians or hospital beds per population. These indicators provide important information on resource availability and inequality but are not designed to compare different manifestations of talent agglomeration. Consequently, whether the same economic, social, and ecological factors have different associations with different dimensions of MHT agglomeration remains unclear. To address this issue, this study uses panel data from 285 prefecture-level cities over 16 consecutive years (2004–2019) to examine these dimension-specific associations.

This paper makes the following contributions. First, it constructs an integrated economic–social–ecological analytical framework that transcends single-dimension. Incorporating squared terms for economic variables, it examines the nonlinear associations of per capita GRP and science and technology expenditure with MHT agglomeration, extending the linear specifications used in most existing studies. Second, it extends our previously developed multidimensional framework to a longitudinal city-level setting, shifting the focus from measurement and spatiotemporal description to the analysis of associated factors. Specifically, we use the four dimensions as parallel outcomes to examine whether economic, social, and ecological characteristics have different associations with different manifestations of agglomeration across 285 cities over 2004–2019.

## 2. Institutional Background

Understanding the factors associated with the spatial allocation of MHT in China’s healthcare sector requires a clear account of how the relevant institutional arrangements have evolved. Since China’s reform and opening-up, changes in the healthcare system and personnel management institutions have profoundly influenced the location choices and mobility patterns of medical and health professionals.

### 2.1. Basic Structure of China’s Healthcare System and Policy Background

The regional allocation of medical and health resources in China has long been uneven, with more resources in the east than in the west and in urban areas than in rural areas. This pattern is closely linked to hospital management institutions and policy priorities. Governments at all levels have introduced a series of supporting measures to develop a four-tier healthcare service system led by national medical centers and supported by regional medical centers at various levels. This system forms a comprehensive healthcare service network spanning the national, provincial, municipal, and county levels [7].

The tax-sharing reform of 1994 established separate fiscal responsibilities for central and local governments. Under this framework, local governments bear most expenditure on public services, including healthcare. Economically developed regions can invest more fiscal resources in building medical institutions and developing their healthcare workforce. Less-developed regions face fiscal constraints, creating a cumulative advantage for stronger regions. In their study of medical resource allocation in Xinjiang, Fu et al. [8] found that the development of the medical and health technical workforce and the level of regional economic development were the dominant factors associated with spatial disparities in medical resource allocation. Medical talent attrition was more pronounced in economically less-developed regions.

Regarding social factors, Li [9] found that economically developed megacities strongly attract medical talent through their accumulated social resources and advanced technologies. Consistent with the Matthew effect, the coexistence of talent agglomeration in higher-level medical institutions and severe talent attrition in lower-level institutions has become increasingly pronounced.

The hospital grading management system, implemented since 1989, classifies hospitals into three tiers and ten grades, with tertiary Grade A hospitals concentrated in provincial capitals and regional central cities. The detailed evaluation criteria for tertiary hospitals require rigorous institutional development, quality control, and resource allocation. These requirements confer institutional and managerial advantages on top-tier institutions relative to lower-grade institutions [10]. Consequently, top-tier hospitals benefit from differentiated policies on fiscal allocations, fee standards, equipment provision, and talent recruitment. While standardizing hospital management, the system has also reinforced the spatial hierarchy of medical resources, directing talent toward medical institutions in large cities with higher administrative status.

### 2.2. Evolution of Medical and Health Talent Policies and Regional Talent Competition

Since the beginning of the 21st century, governments at all levels have increasingly regarded talent as the primary resource for innovation and development. In 2002, the Central Committee of the Communist Party of China proposed the strategy of “strengthening the nation through talent,” and in 2010, it issued the Outline of the National Medium- and Long-Term Talent Development Plan (2010–2020). To address interregional imbalances in medical resource allocation, the state has implemented a series of policy interventions targeting western China and other less-developed regions. These regions commonly face insufficient and unevenly distributed healthcare resources and lower operational efficiency in primary care institutions [11]. From 2012 to 2021, the number of health technicians in primary healthcare institutions nationwide increased from 2.0518 million to 3.3016 million, a total increase of 60.91% [12]. Nevertheless, market forces and institutional constraints continue to limit the effectiveness of policies intended to direct talent toward primary care and less-developed areas.

## 3. Literature Review

### 3.1. Talent Agglomeration and Its Measurement Indicators

The Ministry of Science and Technology issued the National Medium- and Long-Term Medical and Health Talent Development Plan (2010–2020) in 2011. The plan defined medical and health talent as workers who possess certain professional knowledge or specialized skills, engage in creative scientific and technological activities, and contribute to the development of science and technology as well as the economy and society [13]. Scientific and technological talent agglomeration occurs during human capital mobility and migration, as individuals converge in a specific geographic region in response to income, resources, culture, and the environment. This process leads to the rapid concentration of scientific and technological talent in that area. Simon (1998) and Giannetti (2001) argued that human capital agglomeration is a form of spatial concentration in which professionals with different skills cluster to complement one another, improve work efficiency, reduce production costs, and achieve economies of scale, forming the basis of the concept of talent agglomeration [14,15]. Liu et al. (2008) defined scientific and technological talent agglomeration as the movement of scientific and technological talent across different regions or units in response to specific conditions [16]. They described it as a complex form of population mobility that reflects differences in the attractiveness of resources to scientific and technological talent [16].

Several indicators have been used to measure MHT agglomeration. Li et al. (2011) measured the density of scientific and technological human resources using an approach analogous to population density, calculating the ratio of the total number of personnel to the total regional area [17]. Zhao et al. (2012) constructed an indicator system for the agglomeration effects of medical and health talent based on the characteristics of such effects [18]. Chen et al. (2017) selected the number of scientific and technological innovation human resources and the size of the total talent pool as variables for constructing MHT agglomeration indicators [19]. Xiu et al. (2017) drew on the concept of location entropy and introduced the notion of MHT agglomeration equilibrium to evaluate talent agglomeration comprehensively [20]. Building on these approaches, Zhang et al. (2025) established a comprehensive four-dimensional index system—encompassing Talent Agglomeration Density (TAD), Talent Agglomeration Scale (TAS), Talent Agglomeration Intensity (TAI), and Talent Agglomeration Equilibrium (TAE) [21]. Using long-term data from 1982 to 2020, they systematically analyzed the spatiotemporal evolution and regional disparities of MHT in China [21]. This multidimensional framework provides the methodological basis for calculating the core indicators in this study.

### 3.2. Factors Associated with Talent Agglomeration

Like changes in population size, talent agglomeration is influenced by numerous factors. Krugman (1991) proposed the “center–periphery” model of horizontal talent agglomeration [22]. Palivos and Wang (1996) identified knowledge spillover effects, fixed asset investment, internal economies of scale, external economies of scale, government talent policies, and salary levels as the main factors influencing talent agglomeration effects [23]. In their study of the relationship between scientific and technological talent and the regional environment, Rotemberg and Saloner (2000) found that healthy scientific and technological talent competition, incentive mechanisms, and the improvement of relevant laws and regulations play important roles in regional scientific and technological talent agglomeration [24]. Researchers in China have conducted extensive research on the driving factors of talent agglomeration. Li (2012) employed a system dynamics approach to analyze the roles of the industrial economic environment, educational environment, and social and cultural environment in talent agglomeration [25]. Li (2013) reported that differences and changes in the natural environment, economic foundation, political environment, and cultural education affect the overall spatial distribution and spatiotemporal evolution of academicians of the Chinese Academy of Sciences to varying degrees [26]. Examining life satisfaction and factors influencing attrition among scientific and technological professionals, Hou et al. (2014) found that ethnic identity, educational attainment, job satisfaction, and life satisfaction are important factors affecting scientific and technological talent agglomeration [27]. Fang (2016) demonstrated that science and technology is the primary factor continuously influencing the spatial agglomeration of the creative class in Guangdong Province [28]. Zhang (2018) found significant positive associations of economic development and local fiscal investment in education with talent agglomeration at all quantiles [29]. Wage levels and public service provision showed significant positive associations at lower quantiles, whereas urbanization showed a significant positive association at higher quantiles [29]. Huang et al. (2018) found a relatively close relationship between the departure of high-level talent and the acquisition of academic capital, but a weaker association with administrative resources and economic capital [30]. They argued that promoting the rational and orderly mobility of high-level talent requires addressing market failures and issues of public policy legitimacy and anomie [30]. Xu et al. (2019) found that economic development level and scientific research environment are the main factors influencing the interprovincial mobility of scientific and technological talent [31]. Ning et al. (2020) analyzed the state of primary healthcare workforce development in Fujian Province, concluding that some areas face problems in recruitment, training and deployment, and safeguards for staff mobility [32]. They recommended strengthening primary healthcare workforce development [32]. Wang (2020) analyzed medical and health resource agglomeration in 13 cities of Jiangsu Province. Registered nurses, health technicians, licensed physicians, and licensed assistant physicians contributed substantially to resource agglomeration [33]. Wuxi, Nanjing, and Suzhou had relatively high levels of medical resource agglomeration [33].

However, several gaps remain in the literature. First, research on agglomeration mechanisms specific to MHT is relatively scarce. Second, most studies use a single indicator to measure talent agglomeration, making it difficult to capture its multidimensional characteristics, including density, scale, intensity, and equilibrium. This study seeks to address these gaps. Drawing on existing research, it constructs an analytical framework encompassing economic, social, and ecological dimensions. Economic factors provide the foundational conditions for talent agglomeration. Per capita GRP reflects regional economic development, whereas science and technology expenditure characterizes the innovation environment. These factors constitute the economic basis for talent agglomeration. Social factors encompass the provision of healthcare resources and cultural facilities, which directly influence professionals’ quality of life and career development opportunities. Ecological factors include indicators of environmental quality, such as air quality and urban greening.

## 4. Materials and Methods

### 4.1. Variables and Data

#### 4.1.1. Dependent Variables

The dependent variables measure the level of medical and health talent (MHT) agglomeration in China. To obtain consistent and comparable city-level measures over 2004–2019, we used the numbers of licensed physicians and licensed assistant physicians reported in the annual China City Statistical Yearbook. These physicians constitute a core professional group within MHT: they are regulated healthcare personnel directly engaged in diagnosis, treatment, and the delivery of medical services. Their numbers therefore provide a useful indication of changes in local healthcare human resources. Previous city-level studies have also used this measure [34,35]. Given data availability, the number of “licensed (assistant) physicians” published in statistical yearbooks was adopted as a proxy variable for MHT. Drawing on existing studies, we constructed a four-dimensional index system to measure the level of MHT agglomeration.

**Talent Agglomeration Density** (TAD): Defined as the ratio of the MHT count to the total area of a region, reflecting the agglomeration degree of MHT per unit area. A higher value indicates greater regional MHT density, whereas a lower value indicates lower density [17,36]. The calculation formula is as follows:

(1) $$TAD_{it}=\frac{\text{Number}\;\text{of}\;MHT_{it}}{A_{it}}$$

where *i* denotes the city, *t* denotes the year, MHT*_it_* represents the MHT count in city *i* in year *t*, and A*_it_* represents the area of city *i* in year *t*.

**Talent Agglomeration Scale** (TAS): Defined as the ratio of a city’s MHT count to the total MHT count in its province, this indicator reflects the city’s share of provincial MHT. A larger share indicates a larger agglomeration scale in the city, whereas a smaller share indicates a smaller scale [20,37]. The calculation formula is as follows:

(2) $$TAS_{it}=\frac{\text{Number}\;\text{of}\;MHT_{it}}{\text{Number}\;\text{of}\;MHT_{t}}$$

where MHT*_t_* denotes the total MHT count in the province where the city is located in year *t*.

**Talent Agglomeration Intensity** (TAI): Defined as the ratio of the MHT count in a city to its total population, this indicator reflects the concentration of MHT relative to the population in each city. A higher ratio indicates greater agglomeration intensity, whereas a lower ratio indicates lower intensity [20,38]. The calculation formula is as follows:

(3) $$TAI_{it}=\frac{\text{Number}\;\text{of}\;MHT_{it}}{\text{Number}\;\text{of}\;TPOP_{it}}$$

where TPOP*_it_* denotes the total population of city *i* in year *t*.

**Talent Agglomeration Equilibrium** (TAE): Constructed with reference to the concept of location entropy, the location entropy index of MHT reflects the equilibrium degree of MHT agglomeration. A higher TAE indicates a higher degree of MHT agglomeration in the city. When TAE > 1, the city’s MHT equilibrium degree is above the provincial average; when TAE < 1, it is below the provincial average; and when TAE = 1, it equals the provincial average [20,39]. The calculation formula is as follows:

(4) $$TAE_{it}=\frac{\text{Number}\;\text{of}\;MHT_{it}/\text{Number}\;\text{of}\;TPOP_{it}}{\sum_{i=1}^{\lambda }MHT_{it}/\sum_{i=1}^{\lambda }TPOP_{it}}$$

where MHT*_it_* denotes the MHT count in city *i* in year *t*, and TPOP*_it_* denotes the total population of city *i* in year *t*.

#### 4.1.2. Explanatory Variables

Drawing on existing studies and considering data availability, explanatory variables were selected from three dimensions: economic, social, and ecological factors.

Economic factors: Economic factors have been a central research focus in studies of job changes, talent attrition, and attraction at the micro level, as well as interregional mobility and international migration at the macro level. Florida [36] examined the relationship between income and the geographic distribution of talent. In addition to per capita GDP, Zhao et al. [40] used the share of tertiary industry output and R&D expenditure as economic indicators. Hu et al. [41,42,43] examined the influence of industrial agglomeration on MHT agglomeration, using the number of high-tech enterprises as an indicator. Drawing on these studies and considering data availability, this study adopts per capita regional gross domestic product and science and technology expenditure as economic indicators. Their squared terms were also included to test for potential nonlinear relationships.

Social factors: Zhao et al. [40] used the number of healthcare institutions as a medical and health indicator within the social dimension. Satpathy et al. [44] included the impact of healthcare levels on migration intentions in their questionnaire. Li et al. [45] examined associations of public library collections per capita and public library floor space per 10,000 population with MHT agglomeration. This paper adopts Urban hospitals, Urban hospital beds (number of hospital beds per 1000 population), and public library collections as social indicators. These variables reflect access to medical resources, medical service capacity, and public cultural service provision, respectively.

Ecological factors: Florida [36] examined the role of warm climates in attracting talent. Cui et al. [46] adopted climate, air quality, and water bodies as indicators of the natural environment when studying the relationship between urban amenities and talent agglomeration. Yu et al. [47,48] also employed climate, air quality, and water quality as natural amenity factors in their research on urban amenities. Zhang [49] adopted average January and July temperatures, sunshine hours, humidity, the proportion of days with good air quality, and the proportion of park green space area as natural environment indicators when studying urban amenity to attract talent in the United States. Sun et al. [50] treated environmental pollution as an influencing factor in their study of the impact of the environment on talent agglomeration. Zou [51] argued that the more severe the environmental pollution, the lower the degree of talent agglomeration. Drawing on existing research and considering data availability, this study uses industrial SO_2_ emissions, industrial dust emissions, urban green space, and industrial wastewater as ecological indicators potentially associated with MHT agglomeration. Among these, green space area reflects the level of ecological development, while pollutant emissions reflect environmental pressure.

To mitigate heteroscedasticity, all continuous variables were transformed using natural logarithms.

Descriptive statistics for all variables are presented in Table 1.

The descriptive statistics show considerable intercity disparities across all four agglomeration indicators. The mean TAD was 3.281, with a standard deviation of 4.186, and the maximum (57.32) was approximately 17.5 times the mean. These values indicate substantial variation in physician density per unit area and a high concentration of MHT in certain cities. The mean TAS was 10.53, with a standard deviation of 16.60 and maximum of 100, reflecting extreme imbalance in the share of physicians across cities within provinces, with individual cities accounting for a predominant proportion of physician resources. The mean TAI was 32.37 per 10,000 population. The wide gap between the minimum (3.332) and maximum (146.3) indicates that physician numbers relative to population in some cities far exceeded the national average, while others remained at relatively low levels. The mean TAE was 0.972, slightly below the provincial average of 1, with a standard deviation of 0.402, also indicating substantial variation.

Among the economic explanatory variables, the mean log-transformed per capita GRP was 10.58, with a standard deviation of 0.776, indicating pronounced differences in economic development across cities. Mean log-transformed science and technology expenditure was 8.560, with a standard deviation of 2.152, reflecting uneven fiscal investment in science and technology. Among social factors, the mean log-transformed number of urban hospitals was 3.923. Mean urban hospital beds were 6.456 per 1000 population, with a maximum of 25.39, highlighting considerable regional differences in healthcare service capacity. Among ecological factors, the standard deviations of log-transformed industrial SO_2_ emissions and industrial dust emissions both exceeded 1, while that of log-transformed industrial wastewater was 1.178. These statistics indicate similarly pronounced intercity differences in environmental pressure. These characteristics provide a basis for the subsequent heterogeneity analysis. Drawing on existing studies and considering data availability, this study includes per capita GRP and science and technology expenditure, along with their squared terms, as economic indicators. The squared terms allow the analysis to detect potential nonlinear associations that linear specifications would not capture.

#### 4.1.3. Data Sources

This study uses panel data from 285 prefecture-level cities in China spanning 2004–2019. The core indicators cover talent agglomeration and economic, social, and ecological factors. Data were sourced from the China City Statistical Yearbook (2005–2020). Missing values were imputed using linear interpolation, a common practice in studies using Chinese prefecture-level panel data [52,53]. Approximately 689 variable-level observations were interpolated, accounting for about 1.01% of the 68400 data values in the final sample of 4560 city-year observations. The variables most affected were industrial SO_2_ emissions (2.74%), industrial wastewater (2.74%), and public library collections (1.12%). Core variables, including per capita GRP and urban hospital beds, had interpolation rates below 1%. Given the low overall proportion of missing values and their dispersed distribution across cities and years, linear interpolation was considered appropriate for this dataset.

### 4.2. Spatial Autocorrelation Analysis

Tobler noted that “the first Law of geography: everything is related to everything else, but near things are more related than distant things” [54]. Spatial autocorrelation analysis is commonly used to examine whether observations of a variable are statistically related across locations and to explore spatial associations among indicators. This study uses Moran’s I and Moran scatter plots to characterize the spatial distribution of MHT across cities and identify patterns of clustering and dispersion.

#### 4.2.1. Global Spatial Autocorrelation

The global Moran’s I reflects the overall spatial autocorrelation of the study area and is used to determine whether the research objects exhibit spatial autocorrelation relationships as a whole. It is a spatial autocorrelation statistic for the entire study area [55,56,57]. The calculation formula is as follows:

(5) $$I=\frac{n\times \sum_{i=1}^{n}\sum_{j=1}^{n}W_{ij}\left(X_{i}-\bar{X}\right)\left(X_{j}-\bar{X}\right)}{\sum_{i=1}^{n}\sum_{j=1}^{n}W_{ij}\sum_{i=1}^{n}{\left(X_{i}-\bar{X}\right)}^{2}}$$

where i ≠ j, n is the number of research objects; X is the observed value, $\bar{X}$ is the mean of Xi; Wij is the spatial weight matrix between research objects i and j, with a value of 1 for spatially adjacent units and 0 for nonadjacent units. Global Moran’s I ranges from [−1, 1].

Observing the Moran’s I helps to determine the clustering and dispersion states of data in space. If Moran’s I < 0, it indicates that the statistical data exhibit a negative spatial relationship, and the closer Moran’s I is to −1, the stronger the negative correlation. If Moran’s I = 0, it indicates that the statistical data exhibit no significant spatial correlation, and the data are randomly and uniformly distributed in space. If Moran’s I > 0, it indicates that the statistical data exhibit a positive spatial correlation, and the closer Moran’s I is to 1, the stronger the positive correlation.

Because the research question concerns the presence of spatial autocorrelation in either direction, all Moran’s I tests were conducted using two-sided alternatives rather than one-sided tests. A total of 64 tests were performed (four indicators × sixteen years). To account for multiple comparisons, we applied the Benjamini–Hochberg false discovery rate (FDR) correction across all 64 tests. Both raw two-sided *p*-values and FDR-adjusted q-values are reported. Results are interpreted using the FDR-adjusted q-values, with q < 0.05 considered statistically significant.

#### 4.2.2. Local Spatial Autocorrelation

To further examine the distribution patterns of MHT, the local spatial autocorrelation analysis method was employed to identify local clustering. The local Moran’s index aims to explore the spatial distribution between a local unit and its adjacent neighbors. The calculation formula is as follows:

(6) $$I_{i}=\frac{\left(X_{i}-\bar{X}\right)}{\sum_{i=1}^{n}{\left(X_{i}-\bar{X}\right)}^{2}}\sum_{j=1}^{n}W_{ij}\left(X_{i}-\bar{X}\right)$$

where Xt and Xj are the observed values of spatial units t and j, respectively; $\bar{X}$ is the mean of X; Wij is the spatial weight matrix between units i and j.

Local indicators of spatial association (LISA) are used here to identify localized clustering patterns. It should be noted that local LISA statistics capture local spatial heterogeneity or club convergence rather than global spatial dependence. Such time-invariant local club effects are absorbed by city fixed effects in the fixed-effects panel model specification, which further supports the use of an ordinary panel model rather than a spatial econometric model.

### 4.3. Panel Regression Model

The core empirical analysis examines factors associated with MHT agglomeration at the city level in China. Economic, social, and ecological factors are included as explanatory variables to assess their associations with city-level MHT agglomeration. Using panel data from 2004 to 2019, we specify the following econometric model [58]:

(7) $$MHA_{it}=\beta_{0}+\beta_{1}{Econ}_{it}+\beta_{2}{Soc}_{it}+\beta_{3}{Ecol}_{it}+\mu_{it}$$

where *i* denotes each city, and *t* denotes the year. The dependent variable MHA represents the MHT agglomeration level, measured by the four indicators TAD, TAS, TAI, and TAE, respectively. Econ represents economic factors, Soc represents social factors, and Ecol represents ecological factors. *μ_it_* is the random error term.

To examine potential nonlinear associations, the variables in the three dimensions are specified individually, and squared terms for economic factors are incorporated into the following extended model:

(8) $$\begin{array}{cc} MHA_{it} & =\beta_{0}+\beta_{1}Econ1_{it}+\beta_{2}{{Econ1}_{it}}^{2}+\beta_{3}Econ2_{it}+\beta_{4}{{Econ2}_{it}}^{2}\\ & +\beta_{5}Soc1_{it}+\beta_{6}Soc2_{it}+\beta_{7}Soc3_{it}\\ & +\beta_{8}Ecol1_{it}+\beta_{9}Ecol2_{it}+\beta_{10}Ecol3_{it}+\beta_{11}Ecol4_{it}+\mu_{it} \end{array}$$

where *i* denotes the city and *t* denotes the year. The dependent variable MHA represents MHT agglomeration, measured in separate models by TAD, TAS, TAI, and TAE. The explanatory variables are defined as follows. Econ1 denotes per capita gross regional product (GRP); Econ1^2^ denotes the squared term of per capita GRP. Econ2 denotes science and technology expenditure. Econ2^2^ denotes the squared term of science and technology expenditure. Soc1 denotes the number of urban hospitals; Soc2 denotes urban hospital beds per 1000 population; Soc3 denotes public library collections. Ecol1 denotes industrial SO_2_ emissions; Ecol2 denotes industrial dust emissions; Ecol3 denotes urban green space; Ecol4 denotes industrial wastewater. *β_1_* to *β_11_* are the coefficients to be estimated, and *μ_it_* is the random error term.

Compared with existing studies, this model explicitly includes squared terms for economic factors (Econ1^2^ and Econ2^2^) to test potential nonlinear associations of economic development and science and technology investment with talent agglomeration. This specification allows marginal effects to vary with the level of each explanatory variable, capturing differences in the associations of economic factors at different stages of development.

The Hausman test was used to determine the appropriate specification between fixed-effects and random-effects models. The baseline specification includes both city fixed effects and year fixed effects. City fixed effects control for time-invariant city-level heterogeneity, while year fixed effects absorb common temporal shocks that affect all cities in a given year, such as national healthcare reforms, macroeconomic fluctuations, and environmental regulations. Standard errors are clustered at the city level and reported in parentheses.

## 5. Results

### 5.1. Spatial Autocorrelation Analysis

#### 5.1.1. Global Moran’s I

Global Moran’s I tests were conducted for TAD, TAS, TAI, and TAE to assess spatial autocorrelation in MHT agglomeration. Table 2 presents the global Moran’s I values for the four indicators from 2004 to 2019.

Table 2 reports global Moran’s I for TAD, TAS, TAI, and TAE from 2004 to 2019. All tests used two-sided alternatives, and the Benjamini–Hochberg FDR correction was applied across the 64 tests. After correction, none of the Moran’s I values was statistically significant at the 5% level. The smallest two-sided *p*-value was 0.093 for TAI in 2011, corresponding to an FDR-adjusted q-value of approximately 0.974.

The Moran’s I values for the vast majority of years were close to zero, indicating that the spatial distribution of MHT at the city level in China exhibits a generally random and dispersed pattern, with no significant global spatial dependence. This evidence supports the appropriateness of employing an ordinary panel model rather than a spatial econometric model in the subsequent econometric analysis.

#### 5.1.2. Local Moran’s I

**Figure 1:**
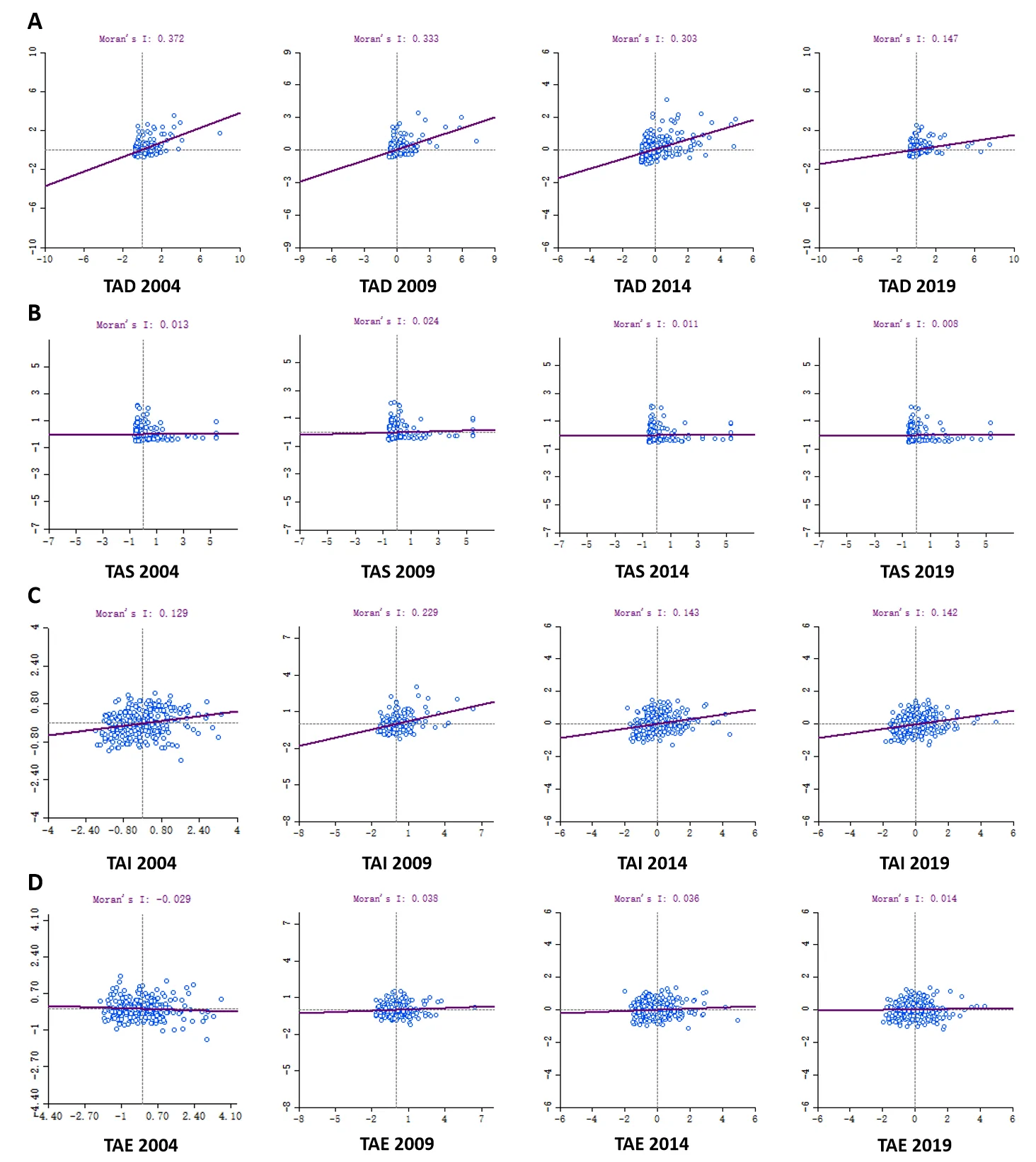
Local Moran scatter plots for TAD, TAS, TAI, and TAE in 2004, 2009, 2014, and 2019. (**A**) TAD; (**B**) TAS; (**C**) TAI; (**D**) TAE.

Although global Moran’s I was not statistically significant, the local indicators of spatial association (LISA) analysis can still reveal spatial club characteristics within specific regions. Figure 1 presents the local Moran scatter plots for TAD, TAS, TAI, and TAE in four representative years: 2004, 2009, 2014, and 2019.

The evolution of the scatter plots over time suggests several patterns. First, the local Moran’s I for TAD declined continuously, from 0.372 in 2004 to 0.147 in 2019. Most cities were located in the first and third quadrants, indicating that higher values tended to be surrounded by higher values and lower values by lower values. This pattern of spatial association in agglomeration density weakened over the sample period.

Second, the local Moran’s I for TAS remained very low, ranging from 0.008 to 0.024 throughout the study period, with no discernible trend. Scatter plot patterns were similar across years, with cities distributed across the first, second, and third quadrants. This suggests a stable pattern of weak spatial association, consistent with the global Moran’s I findings.

Third, the local Moran’s I for TAI followed an inverted V-shaped trajectory, rising from 0.129 in 2004 to 0.229 in 2009 before declining to 0.142 by 2019. Most cities were in the first and third quadrants. The reported spatial autocorrelation for TAI was stronger than that for TAS but weaker than that for TAD, suggesting a limited degree of regional similarity in physician allocation relative to population.

Fourth, the local Moran’s I for TAE evolved from a negative value (−0.029 in 2004) to a value near zero (0.014 in 2019). The weak negative correlation observed in 2004 became positive and close to zero after 2009. Most cities were situated in the first and third quadrants. This transition indicates that the spatial association of MHT equilibrium across cities gradually weakened, with the equilibrium levels of individual cities tending to evolve independently.

The local Moran’s I analysis further corroborates the findings of the global test. The overall degree of spatial agglomeration of MHT is weak and exhibits a further weakening trend. Although TAD maintained a certain degree of positive spatial correlation at the local scale, its intensity continuously declined. This evidence supports the appropriateness of employing an ordinary panel model rather than a spatial econometric model in the subsequent econometric analysis.

### 5.2. Panel Regression

Prior to the panel regression analysis, the Hausman test was employed to determine the appropriate specification between fixed-effects and random-effects models. The Hausman test rejected the null hypothesis at the 1% significance level for all four models, supporting the fixed-effects specification. For the TAS model, the initial Hausman test produced a negative chi-square statistic, which fails to meet the asymptotic assumptions of the test; we therefore re-conducted the test with the sigmamore option and obtained a positive chi-square statistic (chi2 = 252.50, *p* < 0.001), again supporting the fixed-effects specification. The baseline specification includes both city fixed effects and year fixed effects, and standard errors are clustered at the city level. City fixed effects control for time-invariant city-level heterogeneity, while year fixed effects absorb common temporal shocks that affect all cities in a given year, such as national healthcare reforms, macroeconomic fluctuations, and environmental regulations. Standard errors are reported in parentheses.

Model (1) in Table 3 reports the two-way FE regression with TAD as the dependent variable. Among economic factors, per capita GRP was not statistically significant, whereas its squared term was significantly positive at the 5% level, revealing a U-shaped relationship betweenPer capita GRP and TAD; Sci-tech expenditure was significantly negative at the 1% level, while its squared term was significantly positive at the 1% level, revealing a U-shaped relationship between sci-tech expenditure and TAD: when sci-tech expenditure is at a low level, increases in such expenditure are accompanied by decreases in TAD; once the expenditure surpasses the turning point, further increases are accompanied by increases in TAD, marginal effect and turning point was shown in Table 4. Among social factors, Urban hospitals was significantly negative at the 5% level, indicating a negative association with physician density per unit area. Urban hospital beds were significantly positive at the 1% level. Among ecological factors, urban green space was significantly negative at the 10% level, whereas industrial wastewater was significantly positive at the 10% level.

Model (3) reports the two-way FE regression results with TAS as the dependent variable. Among social factors, Urban hospitals was significantly positive at the 1% level, and urban hospital beds were significantly positive at the 5% level. Among ecological factors, only urban green space was significantly positive, at the 10% level, indicating that cities with more green space had a larger share of provincial physicians. All other ecological variables were not statistically significant. Economic factors and most ecological factors were not statistically significant in this model.

Model (5) reports the two-way FE regression results with TAI as the dependent variable. Among economic factors, Sci-tech expenditure squared term was significantly positive at the 5% level, revealing a U-shaped relationship between Sci-tech expenditure and TAI, marginal effect and turning point was shown in Table 4. Among social factors, urban hospital beds were significantly positive at the 1% level. Among ecological factors, industrial wastewater was significantly positive at the 10% level.

Model (7) reports the two-way FE regression results with TAE as the dependent variable. Among economic factors, Sci-tech expenditure squared term was significantly negative at the 10% level, revealing inverted U-shaped relationship between Sci-tech expenditure and TAE, marginal effect and turning point was shown in Table 4. Among social factors, urban hospital beds were significantly positive at the 1% level.

Across the four models, the associations between the explanatory factors and dimensions of MHT agglomeration differed substantially. Among economic factors, the squared term for per capita GRP was significant only in the TAD model (5%). Science and technology expenditure was significantly negative in the TAD model (1%), whereas its squared term was significantly positive in the TAD (1%) and TAI (5%) models and significantly negative in the TAE model (10%). The estimated turning point for per capita GRP (3.69) and those for sci-tech expenditure (8.47, 6.91, and 5.75) all fall within the observed ranges of the corresponding explanatory variables (per capita GRP: −1.763 to 15.68; sci-tech expenditure: 0.693 to 15.53). Thus, these nonlinear patterns occur within the observed sample range rather than being extrapolated beyond the data. Among social factors, urban hospital beds were significantly positive in the TAD (1%), TAS (5%), TAI (1%), and TAE (1%) models, making it the only explanatory variable that remained robust across all dimensions. Urban hospitals was significantly negative in the TAD model (5%) and significantly positive in the TAS model (1%). Among ecological factors, urban green space was significantly negative in the TAD model (10%) and significantly positive in the TAS model (10%). Industrial wastewater was significantly positive in the TAD (10%) and TAI (10%) models.

### 5.3. Robustness and Endogeneity-Related Checks

To assess the reliability of the baseline results, this study employed six methods for robustness and endogeneity-Related testing: winsorization, excluding municipalities, complete-case sensitivity analysis, one-period-lagged explanatory variables, province × year fixed effects, city-specific linear trends. The regression results for each robustness test are reported in Table 5, Table 6, Table 7, Table 8, Table 9 and Table 10, respectively.

#### 5.3.1. Robustness Test 1: Winsorization

To exclude the potential influence of extreme values on the regression results, all continuous variables were winsorized at the 1st and 99th percentiles before re-estimation. Table 5 reports the estimation results after winsorization. In the TAD model, the squared term for per capita GRP was significantly positive at the 10% level, urban hospital beds remained significantly positive at the 1% level, and industrial wastewater was significantly positive at the 10% level. In the TAS model, Urban hospitals and urban hospital beds both remained significantly positive, and urban green space was significantly positive at the 1% level. In the TAI model, per capita GRP was significantly negative at the 1% level, whereas its squared term was significantly positive at the 1% level. Urban hospital beds were significantly positive at the 1% level. In the TAE model, urban hospital beds remained significantly positive at the 1% level.

#### 5.3.2. Robustness Test 2: Excluding Municipalities

Considering that municipalities directly under the central government differ systematically from other cities in terms of administrative hierarchy, resource allocation, and talent agglomeration, which may exert a disproportionate influence on the regression results, the four municipalities of Beijing, Tianjin, Shanghai, and Chongqing were excluded from the sample before re-estimation. Table 6 presents the results. In the TAD model, the squared term for per capita GRP was significantly positive at the 5% level. Science and technology expenditure was significantly negative at the 5% level, whereas its squared term was significantly positive at the 5% level. Urban hospitals was significantly negative at the 5% level, urban hospital beds remained significantly positive at the 1% level, and urban green space was significantly negative at the 10% level. In the TAS model, Urban hospitals and urban hospital beds both remained significantly positive, and urban green space was significantly positive at the 10% level. In the TAI model, the squared term for science and technology expenditure was significantly positive at the 10% level, and urban hospital beds remained significantly positive at the 1% level. In the TAE model, the squared term for science and technology expenditure was significantly negative at the 5% level, and urban hospital beds remained significantly positive at the 1% level.

#### 5.3.3. Robustness Test 3: Complete-Case Sensitivity Analysis

To further examine whether the baseline results are driven by interpolated observations, we conducted a complete-case sensitivity analysis using the original unbalanced panel without any interpolation. Specifically, all city-year observations containing missing values were excluded, and the four baseline two-way fixed-effects models were re-estimated on the remaining fully original observations with city-level clustered standard errors. The results are reported in Table 7.

The core findings remain qualitatively unchanged. In particular, urban hospital beds per 1000 population remains significantly positive across all four dimensions of MHT agglomeration, confirming that the robust positive association between hospital-bed capacity and talent agglomeration is not an artifact of interpolated observations. The nonlinear associations of per capita GRP and sci-tech expenditure are also broadly consistent with the baseline results: per capita GRP exhibits a U-shaped relationship with TAD (linear term: β = −0.3827, *p* < 0.05; squared term: β = 0.0454, *p* < 0.05), and sci-tech expenditure exhibits U-shaped relationships with TAD (linear term: β = −0.5308, *p* < 0.01; squared term: β = 0.0311, *p* < 0.01) and TAI (linear term:β = −1.3180, *p* < 0.05; squared term: β = 0.0784, *p* < 0.05), as well as an inverted U-shaped relationship with TAE (squared term: β = −0.0019, *p* < 0.10). The signs and magnitudes remain consistent with the baseline results. The complete-case sensitivity analysis suggests that the main conclusions are robust to the exclusion of interpolated observations.

#### 5.3.4. Potential Endogeneity Check1: One-Period-Lagged Explanatory Variables

Considering that economic, social, and ecological factors may exert lagged effects on talent agglomeration, all explanatory variables were replaced with their one-period-lagged values before re-estimation to test temporal robustness. Table 8 reports the estimation results with one-period-lagged explanatory variables. In the TAD model, the squared term for per capita GRP was significantly positive at the 5% level. Science and technology expenditure was significantly negative at the 1% level, whereas its squared term was significantly positive at the 5% level. Urban hospitals was significantly negative at the 10% level, and urban hospital beds remained significantly positive at the 1% level. In the TAS model, Urban hospitals and urban hospital beds were both significantly positive at the 10% level, and urban green space was significantly positive at the 5% level. In the TAI model, the linear term for per capita GRP was significantly negative at the 10% level, the squared term for science and technology expenditure was significantly positive at the 5% level, and urban hospital beds remained significantly positive at the 1% level. In the TAE model, Urban hospitals was significantly negative at the 10% level, and urban hospital beds remained significantly positive at the 1% level. These results indicate that the core conclusions of the baseline regression remain valid after accounting for time-lag effects.

#### 5.3.5. Potential Endogeneity Check2: Province × Year Fixed Effects

To provide additional evidence beyond the one-period-lag robustness test, province-by-year fixed effects were included to absorb unobserved time-varying shocks shared by cities within the same province. Table 9 reports the estimation results with province-by-year fixed effects. Per capita GRP was significantly negative at the 10% level in TAD model, Per capita GRP was significantly negative at the 5% level, and the squared term was significantly positive at the 10% level in TAI model. Sci-tech expenditure was significantly negative at the 5% level, with its squared term significantly positive at the 10% level in TAD model, Sci-tech expenditure was significantly positive at the 5% level with its squared term significantly negative at the 1% level in the TAE model. Urban hospitals was significantly negative at the 5% level in the TAD model and significantly positive at the 1% level in the TAS model. Urban hospital beds remained significantly positive at the 1% level in the TAD, TAI, and TAE models and at the 5% level in the TAS model. Urban green space was significantly negative at the 1% level in the TAD model and significantly positive at the 10% level in the TAS model. The remaining variables were not statistically significant.

#### 5.3.6. Potential Endogeneity Check 3: City-Specific Linear Trends

City-specific linear trends were included to account for heterogeneous long-term development trajectories. Table 10 reports the estimation results with city-specific linear trends. Per capita GRP was not statistically significant in most models, whereas its squared term was significantly positive at the 10% level in the TAD model. Science and technology expenditure was significantly positive, and its squared term was significantly negative at the 10% level in the TAS, TAI, and TAE models. Urban hospitals was significantly positive at the 1% level in the TAS model. Urban hospital beds remained significantly positive in all four models, at the 1% level in the TAD, TAI, and TAE models and at the 10% level in the TAS model. Public library collections were significantly negative at the 5% level in the TAI model. Industrial dust emissions were significantly negative at the 1% level in the TAD model, and urban green space was significantly negative at the 10% level in the TAD model. The remaining variables were not statistically significant.

Across these checks, urban hospital beds remained the most robust factor, with consistently positive associations with MHT agglomeration across all four dimensions. The signs of the other core variables were directionally consistent with the baseline regression.

### 5.4. Heterogeneity Tests

Considering the significant disparities across different regions of China in terms of economic development levels, industrial structures, and medical resource allocation, this study divided the full sample into three regional subsamples—eastern, central, and western—based on geographic location, and conducted separate group regressions to examine the regional heterogeneity of various factors on MHT agglomeration. Table 11 presents the regional subgroup regression results.

Table 11 shows the following regional patterns.

In the eastern region, science and technology expenditure was significantly negative at the 5% level and its squared term was significantly positive at the 5% level in the TAD model. In the TAI model, the linear term was significantly negative at the 10% level and the squared term was significantly positive at the 5% level. Among social factors, Urban hospitals was significantly positive at the 1% level in the TAS model. Urban hospital beds were significantly positive at the 1% level in the TAD, TAI, and TAE models, while public library collections were significantly negative at the 10% level in the TAE model. Among ecological factors, industrial dust emissions were significantly positive at the 10% level in the TAE model, and urban green space was significantly positive at the 5% level in the TAS model. Industrial wastewater was significantly negative at the 10% level in the TAS model and at the 1% level in the TAE model.

In the central region, science and technology expenditure was significantly positive at the 5% level and its squared term was significantly negative at the 1% level in the TAE model. Among social factors, Urban hospitals was significantly positive at the 1% level in the TAS model, and urban hospital beds were significantly positive at the 1% level in the TAD, TAI, and TAE models. Among ecological factors, industrial SO_2_ emissions were significantly negative at the 5% level in the TAI model, and industrial wastewater was significantly positive at the 10% level in the TAS model.

In the western region, per capita GRP was significantly negative at the 5% level and its squared term was significantly positive at the 5% level in the TAI model. The squared term for science and technology expenditure was significantly positive at the 5% level in the TAD model. Among social factors, Urban hospitals was significantly negative at the 10% level in the TAD model. Urban hospital beds were significantly positive at the 5% level in the TAD and TAS models and at the 1% level in the TAI and TAE models. Among ecological factors, industrial dust emissions were significantly negative at the 10% level in the TAD model, and urban green space was significantly negative at the 10% level in the TAD model.

Across the three regional subgroup regressions, urban hospital beds maintained significant positive associations in the TAD, TAI, and TAE models in the eastern, central, and western regions. Hospital bed capacity was the only explanatory variable with consistently significant associations across all three regions and multiple dimensions.

## 6. Discussion

The integrated economic, social and ecological analytical framework considered in this study captures fundamental contextual domains relevant to the attraction, distribution, and retention of healthcare professionals across different institutional and healthcare settings. Economic conditions reflect employment opportunities, professional development, and regional resource capacity. Social conditions represent healthcare infrastructure and public services, while ecological conditions capture environmental and residential characteristics that influence urban attractiveness. The specific variables used to represent each dimension may differ across countries according to institutional arrangements, healthcare system characteristics, and data availability. Nevertheless, the multidimensional structure provides a transferable framework for examining healthcare talent agglomeration in different contexts.

First, economic factors exhibited nonlinear associations. The quadratic specification for per capita GRP suggested a U-shaped relationship with TAD, with an estimated turning point of 3.69, whereas its linear and squared terms were not statistically significant in the TAS, TAI, and TAE models. Science and technology expenditure showed quadratic patterns consistent with U-shaped relationships with TAD and TAI, with estimated turning points of 8.47 and 6.91, respectively, and an inverted U-shaped relationship with TAE, with an estimated turning point of 5.75. These estimates suggest nonlinear associations that differ across dimensions of talent agglomeration.

Second, medical resource capacity showed a particularly consistent association with agglomeration. Urban hospital beds were significantly positive in all four baseline models, making hospital bed capacity the only explanatory variable with significant associations across all four dimensions. This variable retained significant positive associations across the robustness tests and additional fixed-effects checks. In the regional analysis, it remained statistically significant in the TAD, TAI, and TAE models across the eastern, central, and western regions. However, the association may be bidirectional, as hospital capacity and physician agglomeration may influence each other. This finding is consistent with the incentives created by the professional title promotion system [59]. Studies of health resource allocation in China indicate that bed provision is not merely a measure of service capacity but also an important basis for allocating medical resources. Structural surpluses or shortages of beds directly affect operational efficiency and the distribution of talent [60]. By considering disease complexity and service efficiency, using measures such as the case mix index (CMI) and bed utilization index, hospitals can evaluate and adjust bed provision. These adjustments influence the allocation of institutional resources, including staffing quotas and senior professional positions [61]. Bed numbers are closely related to approved staffing quotas and the number of senior professional title positions in medical institutions, influencing professionals’ career development prospects and income expectations. Bed provision therefore represents both medical service capacity and a basis for resource allocation that affects promotion opportunities and the quality of research platforms.

Third, ecological associations differed across agglomeration dimensions. Urban green space was negatively associated with TAD and positively associated with TAS, but its associations with TAI and TAE were not statistically significant. This divergence may reflect the dual role of green space in talent location decisions: abundant green space improves urban livability, while cities with more green space often have lower population density, potentially contributing to lower physician density per unit area [62]. Industrial SO_2_ emissions and industrial dust emissions were not statistically significant in the baseline models. Industrial wastewater showed weak positive associations with TAD and TAI, but these were not stable across more stringent specifications. Environmental quality is generally an important component of location attractiveness [63]. Air pollution tends to discourage highly educated workers and professionals from migrating to or locating in affected areas in China [64,65], whereas urban amenities, including green space and public services, are generally associated with greater attractiveness to talent [66]. Accordingly, positive coefficients for industrial emissions should not be interpreted as evidence that pollution attracts MHT. Similarly, the negative coefficient for urban green space in the TAD model does not establish that healthcare professionals prefer fewer green amenities. The industrial emission variables are aggregate measures rather than direct measures of individual pollution exposure and may also proxy the scale and composition of industrial activity. Residual confounding by urban scale, population density, industrial structure, healthcare quality, and other unobserved city characteristics cannot be excluded. Furthermore, TAD, TAS, TAI, and TAE measure different dimensions of agglomeration and have different denominators. Differences in coefficient signs may therefore reflect the statistical properties of the outcomes rather than fundamentally different environmental preferences.

Fourth, the results showed regional heterogeneity. Science and technology expenditure showed quadratic patterns consistent with U-shaped associations with TAD and TAI in the eastern region, an inverted U-shaped association with TAE in the central region, and a positive quadratic association with TAD in the western region. These patterns suggest substantial regional differences in nonlinear associations. Per capita GRP was not statistically significant in most regional models but exhibited a U-shaped association with TAI in the western region, suggesting that its association may vary across stages of development in some regional contexts. These regional differences provide an empirical basis for region-specific policies. High-level talent generally moves toward southeastern China, where a regional talent circulation pattern has begun to develop; central and western regions and northeastern China commonly experience severe talent deficits [30]. In the eastern region, where salaries are competitive, the competition for talent increasingly centers on quality of life. Urban hospital beds remained statistically significant in the TAD, TAI, and TAE models across the eastern, central, and western regions, making hospital bed capacity the only explanatory variable with consistently significant associations across all three regions and multiple dimensions. Existing evidence indicates distinct regional patterns in healthcare resource development: bed supply has grown significantly faster in central and western China than in the eastern region, whereas demand for beds has grown faster in economically developed eastern coastal provinces than in central and western regions [60]. These differences suggest new institutional directions for medical and health talent policies based on regional coordination. Strategic planning should account for both expansion in supply-side capacity and population shifts affecting demand.

Compared with the existing literature, this paper makes two incremental contributions. First, it extends the previously established multidimensional framework for analyzing MHT agglomeration, including density, scale, intensity, and equilibrium, to a longitudinal city-level setting. Second, it integrates economic, social, and ecological dimensions within a single analytical framework, that transcends single-dimension discussions, providing a comprehensive perspective on the complex factors associated with medical talent agglomeration.

This study has several limitations. First, although licensed physicians and licensed assistant physicians represent a core professional group of MHT, the measure does not include nurses, public-health professionals, researchers, or other health personnel. Accordingly, the findings should primarily be interpreted as reflecting physician agglomeration rather than the entire MHT workforce. Furthermore, talent heterogeneity in terms of educational background, professional title, and specialty fields, may obscure differences in the agglomeration mechanisms of sub-groups in healthcare workforce. Second, although the proportion of interpolated observations was small, the use of linear interpolation may still introduce some degree of measurement uncertainty. Third, city-level yearbook indicators and aggregate industrial-emission variables may be subject to measurement error and may not perfectly capture individual-level data. Fourth, time-varying omitted variables cannot be fully eliminated by the observational panel design. Future research can be deepened in the following directions: first, employing micro-level survey data to identify the decision-making mechanisms of individual-level talent location choices; second, conducting heterogeneity analyses by specialty fields (such as clinical medicine, public health, and basic medicine) to enhance the specificity and policy guidance value of the research conclusions; third, future studies should employ stronger identification strategies to evaluate potential causal relationships between MHT agglomeration and its associated factors.

## 7. Conclusions

Using panel data from 285 cities in China spanning 2004 to 2019, this study combined spatial autocorrelation analysis with a two-way fixed-effects panel model to examine economic, social, and ecological factors associated with MHT agglomeration. The main conclusions are as follows:

First, the spatial distribution of MHT at the city level in China exhibits an overall random and dispersed pattern, with insignificant global spatial autocorrelation; however, local club convergence characteristics of high-high and low-low agglomeration exist at the local scale.

Second, economic factors showed nonlinear associations that differed across dimensions of MHT agglomeration. The quadratic specification for per capita GRP suggested a U-shaped association with TAD, whereas science and technology expenditure showed patterns consistent with U-shaped associations with TAD and TAI and an inverted U-shaped association with TAE. Thus, the associations of economic development differed across dimensions of talent agglomeration.

Third, among social factors, urban hospital beds showed the most robust positive associations with MHT agglomeration. These associations remained significant across all model specifications and across multiple outcomes in the regional heterogeneity analyses.

Fourth, ecological factors showed weaker associations that differed across agglomeration dimensions. Urban green space was negatively associated with TAD and positively associated with TAS, whereas associations involving industrial pollution indicators were generally not statistically significant or were unstable across model specifications.

Fifth, factors associated with talent agglomeration exhibit significant regional heterogeneity. Urban hospital beds remained consistently significant across all three regions, reinforcing their role as factors associated with MHT agglomeration irrespective of regional development levels. Science and technology expenditure showed distinct nonlinear patterns in the eastern, central, and western regions, whereas per capita GRP showed a U-shaped association with TAI only in the western region.

## 8. Policy Implications

Based on these empirical findings, this study proposes the following policy recommendations.

Medical resource capacity should be a core component of regional talent policy. Urban hospital beds were the most robust variable across the tests in this study, showing significant positive associations in all four baseline models and retaining these associations across the robustness tests and checks for potential endogeneity. The regional analysis also showed significant positive associations across multiple agglomeration outcomes, indicating that medical infrastructure remains a priority at the current stage. Given these robust associations, governments at all levels should incorporate improvements of medical resource capacity into their broader planning for talent development.

A mechanism for regularly reviewing and adjusting talent policies should be established. This study found nonlinear associations between economic factors and MHT agglomeration that differed across dimensions. Per capita GRP and science and technology expenditure showed different quadratic patterns consistent with U-shaped or inverted U-shaped associations across the four agglomeration dimensions. Regular monitoring and evaluation of factors associated with talent agglomeration are recommended. Policy priorities should be reviewed as economic development and science and technology investment approach or cross the estimated turning points.

## Figures and Tables

**Table 1:** Descriptive statistics of panel data.

VARIABLES			Sample Size	Mean	Standard Deviation	Min	Max
Dependent variables	TAD		4560	3.281	4.186	0.0254	57.32
	TAS		4560	10.53	16.60	0.314	100
	TAI		4560	32.37	15.50	3.332	146.3
	TAE		4560	0.972	0.402	0.169	4.245
Independent variables	Economic factors	Per capita GRP	4560	10.58	0.776	−1.763	15.68
		Per capita GRP squared	4560	112.4	15.90	3.108	245.7
		Sci-tech expenditure	4560	8.560	2.152	0.693	15.53
		Sci-tech expenditure squared	4560	77.90	37.46	0.480	241.2
	Social factors	Urban hospitals	4560	3.923	0.803	1.386	7.479
		Urban hospital beds	4560	6.456	2.876	0.0301	25.39
		Public library collections	4560	5.890	1.476	0.693	11.25
	Ecological factors	Industrial SO_2_ emissions	4560	10.28	1.227	0.405	13.43
		Industrial dust emissions	4560	9.664	1.141	3.555	15.46
		Urban green space	4560	8.013	1.117	3.332	12.03
		Industrial wastewater	4560	8.256	1.178	0.693	11.48

**Table 2:** Global Moran’s I of TAD, TAS, TAI, and TAE (2004–2019).

Year	TAD Moran’s I	TAD Z	TAD p	TAS Moran’s I	TAS Z	TAS p	TAI Moran’s I	TAI Z	TAI p	TAE Moran’s I	TAE Z	TAE p
2004	0.008	1.485	0.138	−0.004	−0.111	0.912	−0.010	−0.830	0.407	−0.010	−0.797	0.425
2005	0.003	0.787	0.431	−0.005	−0.168	0.867	−0.002	0.142	0.887	−0.010	−0.819	0.413
2006	−0.002	0.239	0.811	−0.007	−0.497	0.619	−0.008	−0.535	0.593	−0.007	−0.488	0.626
2007	−0.001	0.335	0.737	−0.004	−0.114	0.909	−0.004	−0.089	0.929	−0.005	−0.193	0.847
2008	0.001	0.599	0.549	−0.006	−0.300	0.764	−0.009	−0.731	0.465	−0.008	−0.570	0.568
2009	0.000	0.493	0.622	−0.007	−0.500	0.617	−0.001	0.352	0.725	0.000	0.473	0.636
2010	0.000	0.553	0.580	−0.007	−0.499	0.618	−0.006	−0.307	0.759	−0.008	−0.625	0.532
2011	0.000	0.458	0.647	−0.009	−0.639	0.523	0.010	1.678	0.093	0.008	1.433	0.152
2012	−0.001	0.374	0.709	−0.009	−0.725	0.469	0.001	0.584	0.559	0.003	0.767	0.443
2013	−0.003	0.018	0.986	−0.008	−0.569	0.569	−0.008	−0.564	0.572	−0.006	−0.250	0.802
2014	−0.002	0.249	0.803	−0.006	−0.341	0.733	−0.014	−1.250	0.211	−0.013	−1.126	0.260
2015	0.007	1.347	0.178	−0.004	−0.106	0.916	−0.008	−0.571	0.568	−0.012	−1.021	0.307
2016	0.002	0.708	0.479	−0.003	0.051	0.959	−0.005	−0.190	0.849	−0.005	−0.173	0.862
2017	−0.008	−0.539	0.590	−0.006	−0.274	0.784	−0.005	−0.206	0.836	−0.008	−0.499	0.618
2018	−0.002	0.161	0.872	−0.005	−0.164	0.870	−0.006	−0.322	0.748	−0.008	−0.530	0.596
2019	−0.003	0.066	0.948	−0.005	−0.155	0.877	−0.006	−0.355	0.723	−0.009	−0.639	0.523

All 64 Moran’s I tests (4 indicators × 16 years) were conducted using two-sided alternatives. FDR-adjusted q-values were computed using the Benjamini–Hochberg procedure across all 64 tests. A q-value below 0.05 is considered statistically significant. None of the q-values is below 0.05.

**Table 3:** Panel regression results.

VARIABLES			TAD		TAS		TAI		TAE	
			(1) FE	(2) RE	(3) FE	(4) RE	(5) FE	(6) RE	(7) FE	(8) RE
Econ	Econ1	Per capita GRP	−0.266	−0.232	−0.012	−0.013	−1.456	−1.894 **	−0.001	−0.009
			(0.178)	(0.181)	(0.158)	(0.140)	(1.092)	(0.921)	(0.022)	(0.034)
	Econ1^2^	Per capita GRP squared	0.036 **	0.034 **	−0.012	−0.010	0.086	0.173 **	0.001	0.003
			(0.017)	(0.016)	(0.013)	(0.012)	(0.072)	(0.069)	(0.002)	(0.002)
	Econ2	Sci-tech expenditure	−0.542 ***	−0.515 ***	0.072	0.049	−1.023	−1.524 **	0.023	0.013
			(0.197)	(0.197)	(0.132)	(0.132)	(0.646)	(0.638)	(0.019)	(0.017)
	Econ2^2^	Sci-tech expenditure squared	0.032 ***	0.034 ***	0.000	0.003	0.074 **	0.108 ***	−0.002 *	−0.001
			(0.012)	(0.012)	(0.008)	(0.008)	(0.036)	(0.038)	(0.001)	(0.001)
Soc	Soc1	Urban hospitals	−0.305 **	−0.227 *	0.721 ***	0.786 ***	0.498	0.476	−0.005	−0.005
			(0.144)	(0.129)	(0.171)	(0.173)	(0.723)	(0.640)	(0.019)	(0.017)
	Soc2	Urban hospital beds	0.622 ***	0.631 ***	0.126 **	0.130 **	3.073 ***	3.265 ***	0.070 ***	0.076 ***
			(0.122)	(0.118)	(0.055)	(0.055)	(0.319)	(0.270)	(0.009)	(0.008)
	Soc3	Public library collections	0.136	0.180	0.019	0.063	−0.272	−0.085	−0.008	−0.001
			(0.119)	(0.114)	(0.068)	(0.070)	(0.334)	(0.316)	(0.009)	(0.009)
Ecol	Ecol1	Industrial SO_2_ emissions	−0.135	−0.111	0.061	0.078	−0.485	−0.246	0.007	0.009
			(0.135)	(0.128)	(0.079)	(0.081)	(0.367)	(0.316)	(0.011)	(0.010)
	Ecol2	Industrial dust emissions	−0.109	−0.104	−0.027	−0.026	0.184	0.108	−0.000	−0.004
			(0.075)	(0.072)	(0.084)	(0.083)	(0.393)	(0.367)	(0.011)	(0.010)
	Ecol3	Urban green space	−0.198 *	−0.100	0.259 *	0.340 **	−0.206	0.331	−0.002	0.025
			(0.109)	(0.104)	(0.136)	(0.143)	(0.631)	(0.618)	(0.020)	(0.021)
	Ecol4	Industrial wastewater	0.178 *	0.253 ***	0.050	0.061	0.717 *	0.463	0.000	0.007
			(0.102)	(0.092)	(0.080)	(0.080)	(0.404)	(0.318)	(0.012)	(0.010)
Constant			3.864 **	1.283	5.099 ***	3.540 *	17.762 **	9.893 **	0.469 **	0.072
			(1.825)	(1.655)	(1.676)	(1.808)	(7.975)	(5.037)	(0.214)	(0.206)
Observations			4560	4560	4560	4560	4560	4560	4560	4560
R-squared			0.190		0.041		0.455		0.177	
Number of city_id			285	285	285	285	285	285	285	285
Year FE			YES	YES	YES	YES	YES	YES	YES	YES
City FE			YES	NO	YES	NO	YES	NO	YES	NO

Columns (1), (3), (5), (7) report two-way fixed-effects estimates (city and year fixed effects). Columns (2), (4), (6), (8) report random-effects estimates for city effects with year fixed effects. Standard errors are clustered at the city level and reported in parentheses. Year dummies are included but not reported; 2004 is the reference year. The Hausman test, conducted on specifications without year fixed effects, rejects the null hypothesis at the 1% level, supporting the fixed-effects specification. * *p* < 0.1, ** *p* < 0.05, *** *p* < 0.01.

**Table 4:** Marginal effect and turning point.

Outcome	β1	β2	Turning Point	ME at Lower Bound	ME at Upper Bound	Pattern
Per capita GRP→TAD	−0.266	0.036	3.69	−0.393	+0.863	U-shaped
Sci-tech expenditure→TAD	−0.542	0.032	8.47	−0.498	+0.452	U-shaped
Sci-tech expenditure→TAI	−1.023	0.074	6.91	−0.920	+1.275	U-shaped
Sci-tech expenditure→TAE	+0.023	−0.002	5.75	+0.020	−0.039	Inverted U-shaped

**Table 5:** Robustness test 1: Winsorization.

Variables			TAD	TAS	TAI	TAE
Econ	Econ1	Per capita GRP	−3.1563	−1.1186	−28.9672 ***	0.2697
			(2.0045)	(2.0686)	(8.3963)	(0.2449)
	Econ1^2^	Per capita GRP squared	0.1859 *	0.0337	1.4068 ***	−0.0114
			(0.1008)	(0.0959)	(0.4000)	(0.0115)
	Econ2	Sci-tech expenditure	−0.4162	0.1042	−0.0897	0.0044
			(0.2542)	(0.1747)	(0.7985)	(0.0240)
	Econ2^2^	Sci-tech expenditure squared	0.0211	−0.0022	0.0047	−0.0007
			(0.0144)	(0.0109)	(0.0454)	(0.0014)
Soc	Soc1	Urban hospitals	−0.2416	0.7605 ***	0.7645	−0.0001
			(0.1600)	(0.1763)	(0.7790)	(0.0198)
	Soc2	Urban hospital beds	0.6244 ***	0.1396 **	3.1692 ***	0.0735 ***
			(0.1113)	(0.0631)	(0.3353)	(0.0102)
	Soc3	Public library collections	0.1364	0.0010	−0.3986	−0.0108
			(0.1230)	(0.0678)	(0.3179)	(0.0086)
Ecol	Ecol1	Industrial SO_2_ emissions	−0.1744	0.1189	−0.3280	0.0126
			(0.1623)	(0.0894)	(0.4454)	(0.0131)
	Ecol2	Industrial dust emissions	−0.1228	−0.0667	−0.0683	−0.0054
			(0.0871)	(0.0940)	(0.4413)	(0.0124)
	Ecol3	Urban green space	−0.2060	0.3783 ***	0.1937	0.0084
			(0.1263)	(0.1238)	(0.5282)	(0.0172)
	Ecol4	Industrial wastewater	0.2005 *	0.0150	0.6233	−0.0003
			(0.1135)	(0.0828)	(0.4292)	(0.0133)
Control variables			Controlled	Controlled	Controlled	Controlled
City fixed effect			Yes	Yes	Yes	Yes
Year fixed effect			Yes	Yes	Yes	Yes
Observations			4560	4560	4560	4560

All models include city and year fixed effects. Standard errors are clustered at the city level and reported in parentheses. * *p* < 0.10, ** *p* < 0.05, *** *p* < 0.01.

**Table 6:** Robustness test 2: Excluding Municipalities.

Variables			TAD	TAS	TAI	TAE
Econ	Econ1	Per capita GRP	−0.3011	−0.0057	−1.6745	−0.0038
			(0.1882)	(0.1601)	(1.1442)	(0.0219)
	Econ1^2^	Per capita GRP squared	0.0402 **	−0.0122	0.1085	0.0015
			(0.0183)	(0.0131)	(0.0738)	(0.0017)
	Econ2	Sci-tech expenditure	−0.5071 **	0.0689	−0.8532	0.0300
			(0.2105)	(0.1422)	(0.6302)	(0.0190)
	Econ2^2^	Sci-tech expenditure squared	0.0301 **	0.0003	0.0634 *	−0.0022 **
			(0.0129)	(0.0091)	(0.0344)	(0.0011)
Soc	Soc1	Urban hospitals	−0.2974 **	0.7232 ***	0.5325	−0.0053
			(0.1441)	(0.1718)	(0.7278)	(0.0193)
	Soc2	Urban hospital beds	0.6221 ***	0.1257 **	3.0756 ***	0.0707 ***
			(0.1218)	(0.0555)	(0.3195)	(0.0093)
	Soc3	Public library collections	0.1418	0.0190	−0.2481	−0.0076
			(0.1190)	(0.0687)	(0.3341)	(0.0088)
Ecol	Ecol1	Industrial SO_2_ emissions	−0.1161	0.0620	−0.3626	0.0086
			(0.1376)	(0.0804)	(0.3593)	(0.0112)
	Ecol2	Industrial dust emissions	−0.1122	−0.0269	0.1700	−0.0006
			(0.0750)	(0.0841)	(0.3944)	(0.0113)
	Ecol3	Urban green space	−0.2006 *	0.2627 *	−0.1698	−0.0033
			(0.1104)	(0.1375)	(0.6393)	(0.0200)
	Ecol4	Industrial wastewater	0.1676	0.0486	0.6430	0.0001
			(0.1029)	(0.0802)	(0.4061)	(0.0124)
Control variables			Controlled	Controlled	Controlled	Controlled
City fixed effect			Yes	Yes	Yes	Yes
Year fixed effect			Yes	Yes	Yes	Yes
Observations			4496	4496	4496	4496

All models include city and year fixed effects. Standard errors are clustered at the city level and reported in parentheses. * *p* < 0.10, ** *p* < 0.05, *** *p* < 0.01.

**Table 7:** Robustness test 3: Complete-case sensitivity analysis.

Variables			TAD	TAS	TAI	TAE
Econ	Econ1	Per capita GRP	−0.3827 **	0.1567	−1.1840	−0.0007
			(0.1839)	(0.1192)	(0.9514)	(0.0272)
	Econ1^2^	Per capita GRP squared	0.0454 **	−0.0216 *	0.0775	0.0015
			(0.0191)	(0.0128)	(0.0788)	(0.0022)
	Econ2	Sci-tech expenditure	−0.5308 ***	0.0382	−1.3180 **	0.0168
			(0.1926)	(0.1379)	(0.6168)	(0.0181)
	Econ2^2^	Sci-tech expenditure squared	0.0311 ***	0.0002	0.0784 **	−0.0019 *
			(0.0116)	(0.0087)	(0.0348)	(0.0010)
Soc	Soc1	Urban hospitals	−0.3169 **	0.7251 ***	0.3086	−0.0064
			(0.1434)	(0.1751)	(0.6252)	(0.0172)
	Soc2	Urban hospital beds	0.6622 ***	0.1322 **	3.1690 ***	0.0720 ***
			(0.1293)	(0.0571)	(0.3179)	(0.0096)
	Soc3	Public library collections	0.1362	0.0281	−0.1012	−0.0080
			(0.1187)	(0.0715)	(0.3387)	(0.0092)
Ecol	Ecol1	Industrial SO_2_ emissions	−0.1420	0.0377	−0.5503	0.0041
			(0.1420)	(0.0826)	(0.3574)	(0.0109)
	Ecol2	Industrial dust emissions	−0.1100	−0.0521	0.1106	−0.0020
			(0.0785)	(0.0830)	(0.3607)	(0.0109)
	Ecol3	Urban green space	−0.1649	0.3545 ***	−0.0188	0.0141
			(0.1050)	(0.1314)	(0.5643)	(0.0185)
	Ecol4	Industrial wastewater	0.1872 *	0.0454	0.6918	−0.0009
			(0.1092)	(0.0759)	(0.4289)	(0.0130)
Control variables			Controlled	Controlled	Controlled	Controlled
City fixed effect			Yes	Yes	Yes	Yes
Year fixed effect			Yes	Yes	Yes	Yes
Observations			4307	4309	4309	4309

All models include city and year fixed effects. Standard errors are clustered at the city level and reported in parentheses. * *p* < 0.10, ** *p* < 0.05, *** *p* < 0.01.

**Table 8:** Potential Endogeneity Check1: One-Period-Lagged Explanatory Variables.

Variables			TAD	TAS	TAI	TAE
Econ	Econ1	Per capita GRP	−0.2844	−0.0430	−1.6094 *	−0.0111
			(0.2112)	(0.1401)	(0.9704)	(0.0240)
	Econ1^2^	Per capita GRP squared	0.0408 **	−0.0091	0.1107	0.0024
			(0.0203)	(0.0114)	(0.0705)	(0.0018)
	Econ2	Sci-tech expenditure	−0.4447 ***	0.0037	−1.1311	0.0149
			(0.1674)	(0.1356)	(0.6962)	(0.0207)
	Econ2^2^	Sci-tech expenditure squared	0.0282 **	0.0060	0.0878 **	−0.0013
			(0.0111)	(0.0087)	(0.0370)	(0.0011)
Soc	Soc1	Urban hospitals	−0.2265 *	0.2255 *	−0.5226	−0.0295 *
			(0.1340)	(0.1358)	(0.5746)	(0.0163)
	Soc2	Urban hospital beds	0.4996 ***	0.0823 *	1.9328 ***	0.0398 ***
			(0.1059)	(0.0497)	(0.2801)	(0.0078)
	Soc3	Public library collections	0.0898	0.0502	−0.2462	−0.0067
			(0.1052)	(0.0695)	(0.3660)	(0.0095)
Ecol	Ecol1	Industrial SO_2_ emissions	−0.0969	0.1206	0.0753	0.0156
			(0.1317)	(0.0925)	(0.4071)	(0.0115)
	Ecol2	Industrial dust emissions	−0.0989	−0.0502	0.1314	0.0030
			(0.0810)	(0.0765)	(0.3677)	(0.0104)
	Ecol3	Urban green space	−0.1924	0.3326 **	0.0259	0.0028
			(0.1189)	(0.1449)	(0.7175)	(0.0205)
	Ecol4	Industrial wastewater	0.0709	−0.0233	0.2211	−0.0090
			(0.0946)	(0.0858)	(0.4205)	(0.0134)
Control variables			Controlled	Controlled	Controlled	Controlled
City fixed effect			Yes	Yes	Yes	Yes
Year fixed effect			Yes	Yes	Yes	Yes
Observations			4275	4275	4275	4275

All models include city and year fixed effects. Standard errors are clustered at the city level and reported in parentheses.* *p* < 0.10, ** *p* < 0.05, *** *p* < 0.01.

**Table 9:** Potential Endogeneity Check2: Province × Year Fixed Effects.

Variables			TAD	TAS	TAI	TAE
Econ	Econ1	Per capita GRP	−0.2992 *	0.0130	−1.6689 **	−0.0157
			(0.1744)	(0.1605)	(0.7846)	(0.0380)
	Econ1^2^	Per capita GRP squared	0.0306	−0.0132	0.0980 *	0.0037
			(0.0189)	(0.0144)	(0.0585)	(0.0025)
	Econ2	Sci-tech expenditure	−0.5720 **	0.0440	−0.7600	0.0563 **
			(0.2605)	(0.2112)	(0.6370)	(0.0231)
	Econ2^2^	Sci-tech expenditure squared	0.0318 *	0.0032	0.0484	−0.0041 ***
			(0.0172)	(0.0139)	(0.0360)	(0.0013)
Soc	Soc1	Urban hospitals	−0.3462 **	0.8656 ***	−0.0100	−0.0125
			(0.1474)	(0.1873)	(0.6707)	(0.0207)
	Soc2	Urban hospital beds	0.6213 ***	0.1487 **	3.0992 ***	0.0833 ***
			(0.1277)	(0.0605)	(0.3132)	(0.0098)
	Soc3	Public library collections	0.1056	0.0251	−0.4491	−0.0130
			(0.1095)	(0.0763)	(0.3308)	(0.0095)
Ecol	Ecol1	Industrial SO_2_ emissions	−0.1423	0.0848	−0.2525	0.0111
			(0.1545)	(0.0951)	(0.3750)	(0.0124)
	Ecol2	Industrial dust emissions	−0.0514	−0.0168	−0.0863	−0.0078
			(0.0794)	(0.1074)	(0.4450)	(0.0137)
	Ecol3	Urban green space	−0.3255 ***	0.3214 *	−0.4535	−0.0045
			(0.1170)	(0.1772)	(0.6551)	(0.0210)
	Ecol4	Industrial wastewater	0.0395	0.0786	0.3303	−0.0063
			(0.1001)	(0.1042)	(0.4586)	(0.0143)
Control variables			Controlled	Controlled	Controlled	Controlled
Province × Year FE			Yes	Yes	Yes	Yes
City fixed effect			Yes	Yes	Yes	Yes
Observations			4560	4560	4560	4560

All models include city fixed effects and province-by-year fixed effects. Standard errors are clustered at the city level and are reported in parentheses. * *p* < 0.10, ** *p* < 0.05, *** *p* < 0.01.

**Table 10:** Potential Endogeneity Check 3: City-Specific Linear Trends.

Variables			TAD	TAS	TAI	TAE
Econ	Econ1	Per capita GRP	−0.1416	0.0181	−0.2433	−0.0047
			(0.1627)	(0.1000)	(0.5072)	(0.0126)
	Econ1^2^	Per capita GRP squared	0.0256 *	−0.0080	0.0432	0.0007
			(0.0138)	(0.0119)	(0.0598)	(0.0015)
	Econ2	Sci-tech expenditure	0.0608	0.2906 *	2.0841 ***	0.0561 **
			(0.1592)	(0.1553)	(0.7854)	(0.0260)
	Econ2^2^	Sci-tech expenditure squared	0.0038	−0.0207 *	−0.1074 *	−0.0032 *
			(0.0124)	(0.0114)	(0.0561)	(0.0018)
Soc	Soc1	Urban hospitals	−0.2150	0.6830 ***	0.7449	0.0015
			(0.1892)	(0.1829)	(0.9141)	(0.0226)
	Soc2	Urban hospital beds	0.5278 ***	0.1281 *	2.9292 ***	0.0722 ***
			(0.1290)	(0.0682)	(0.4247)	(0.0123)
	Soc3	Public library collections	−0.0366	−0.0522	−0.7138 **	−0.0095
			(0.0624)	(0.0504)	(0.2889)	(0.0071)
Ecol	Ecol1	Industrial SO_2_ emissions	0.0103	−0.1173	−0.4482	−0.0077
			(0.0972)	(0.0736)	(0.3646)	(0.0105)
	Ecol2	Industrial dust emissions	−0.2752 ***	0.0331	−0.6376	−0.0115
			(0.0881)	(0.1049)	(0.4974)	(0.0135)
	Ecol3	Urban green space	−0.1244 *	−0.0359	−0.7846	−0.0161
			(0.0752)	(0.1545)	(0.6715)	(0.0190)
	Ecol4	Industrial wastewater	0.1492	−0.0112	0.3792	0.0103
			(0.1029)	(0.0949)	(0.5160)	(0.0147)
Control variables			Controlled	Controlled	Controlled	Controlled
City fixed effect			Yes	Yes	Yes	Yes
City-specific linear trends			Yes	Yes	Yes	Yes
Observations			4560	4560	4560	4560

All models include city fixed effects and city-specific linear trends. Standard errors are clustered at the city level and are reported in parentheses. * *p* < 0.10, ** *p* < 0.05, *** *p* < 0.01.

**Table 11:** Heterogeneity Tests: Regional Subgroups.

VARIABLES			Eastern Region				Central Region				Western Region			
			TAD	TAS	TAI	TAE	TAD	TAS	TAI	TAE	TAD	TAS	TAI	TAE
Econ	Econ1	Per capita GRP	−0.0959	1.1823	3.6938	0.4049	−0.0187	−0.0474	−0.5599	−0.0082	−0.6344	−2.1721	−18.7642 **	0.3249
			(3.3468)	(1.9501)	(14.3161)	(0.2691)	(0.1622)	(0.1365)	(0.7270)	(0.0272)	(1.5387)	(2.4779)	(9.0925)	(0.2466)
	Econ1^2^	Per capita GRP squared	0.0609	−0.0648	−0.0757	−0.0164	0.0059	−0.0026	0.0744	0.0021	0.0442	0.0750	0.8060 **	−0.0114
			(0.1563)	(0.0846)	(0.6754)	(0.0122)	(0.0189)	(0.0161)	(0.0904)	(0.0029)	(0.0733)	(0.1007)	(0.3959)	(0.0100)
	Econ2	Sci-tech expenditure	−1.3478 **	0.1080	−3.1136 *	0.0196	−0.1594	−0.3617	0.4892	0.0648 **	−0.4157	0.3099	0.6416	0.0339
			(0.5957)	(0.3093)	(1.6772)	(0.0428)	(0.4678)	(0.2658)	(0.9554)	(0.0279)	(0.2865)	(0.2812)	(0.9731)	(0.0331)
	Econ2^2^	Sci-tech expenditure squared	0.0731 **	−0.0005	0.1992 **	−0.0009	0.0034	0.0240	−0.0391	−0.0053 ***	0.0342 **	−0.0165	0.0020	−0.0012
			(0.0342)	(0.0181)	(0.0931)	(0.0023)	(0.0319)	(0.0179)	(0.0511)	(0.0015)	(0.0139)	(0.0220)	(0.0664)	(0.0023)
Soc	Soc1	Urban hospitals	−0.2669	0.9315 ***	0.3758	−0.0102	−0.4602	1.0917 ***	1.9131	0.0049	−0.1856 *	0.3765	−1.1314	−0.0234
			(0.2976)	(0.2349)	(0.8724)	(0.0255)	(0.2884)	(0.3560)	(1.6695)	(0.0469)	(0.1090)	(0.2734)	(0.8159)	(0.0263)
	Soc2	Urban hospital beds	0.9154 ***	0.0971	3.7327 ***	0.0747 ***	0.5001 ***	0.1127	2.9558 ***	0.0781 ***	0.4601 **	0.1683 **	2.7428 ***	0.0650 ***
			(0.2302)	(0.0892)	(0.4321)	(0.0094)	(0.1465)	(0.1133)	(0.6964)	(0.0225)	(0.2191)	(0.0696)	(0.3169)	(0.0092)
	Soc3	Public library collections	0.3033	−0.0493	−0.9131	−0.0284 *	−0.0504	0.0122	−0.2954	−0.0173	0.1078	0.1166	−0.1005	0.0074
			(0.2655)	(0.1126)	(0.6100)	(0.0151)	(0.1225)	(0.0854)	(0.5410)	(0.0152)	(0.2162)	(0.1489)	(0.6263)	(0.0161)
Ecol	Ecol1	Industrial SO_2_ emissions	−0.0255	−0.0274	−0.7611	−0.0224	−0.0464	−0.0549	−1.1755 **	0.0130	−0.3077	0.2390	0.6323	0.0328
			(0.1393)	(0.0869)	(0.5300)	(0.0140)	(0.1523)	(0.1472)	(0.5898)	(0.0207)	(0.3440)	(0.1752)	(0.7272)	(0.0221)
	Ecol2	Industrial dust emissions	−0.0252	0.0126	0.7524	0.0280 *	−0.1572	−0.0815	−0.9530	−0.0273	−0.1964 *	−0.0236	−0.2183	−0.0174
			(0.1520)	(0.0920)	(0.5344)	(0.0154)	(0.1633)	(0.1892)	(0.8253)	(0.0245)	(0.0997)	(0.1398)	(0.6015)	(0.0170)
	Ecol3	Urban green space	−0.3078	0.3923 **	0.3521	0.0002	0.1861	0.3170	0.3545	0.0289	−0.4873 *	0.2040	−0.9179	−0.0277
			(0.2690)	(0.1588)	(0.8478)	(0.0243)	(0.1620)	(0.2257)	(0.6660)	(0.0272)	(0.2498)	(0.2440)	(1.1028)	(0.0351)
	Ecol4	Industrial wastewater	0.2637	−0.1881 *	−0.6252	−0.0493 ***	−0.0187	0.2796 *	0.5978	0.0160	0.2340	−0.0061	1.0570	0.0162
			(0.2431)	(0.1095)	(0.5979)	(0.0138)	(0.1684)	(0.1519)	(0.7246)	(0.0222)	(0.1578)	(0.1491)	(0.6695)	(0.0205)
Observations			1600	1600	1600	1600	1600	1600	1600	1600	1360	1360	1360	1360
adjusted R-squared			0.2230	0.0658	0.6387	0.2935	0.1678	0.0466	0.3647	0.1671	0.2285	0.0109	0.4322	0.1573

All models include city and year fixed effects. Standard errors are clustered at the city level and reported in parentheses. * *p* < 0.10, ** *p* < 0.05, *** *p* < 0.01.

## Data Availability

The dataset can be accessed through the printed yearbooks: China City Statistical Yearbook (2005–2020). The dataset is also available from the corresponding author upon reasonable request.

## Abbreviations

The following abbreviations are used in this manuscript:

MHT	Medical and health talent
TAD	Talent agglomeration density
TAS	Talent agglomeration scale
TAI	Talent agglomeration intensity
TAE	Talent agglomeration equilibrium
